# The prognostic value of pretreatment tumor apparent diffusion coefficient values in nasopharyngeal carcinoma

**DOI:** 10.1186/s12885-017-3658-x

**Published:** 2017-10-11

**Authors:** Dan-Fang Yan, Wen-Bao Zhang, Shan-Bao Ke, Feng Zhao, Sen-Xiang Yan, Qi-Dong Wang, Li-Song Teng

**Affiliations:** 1 Department of Radiation Oncology, the First Affiliated Hospital, College of Medicine, Zhejiang University, 79 Qingchun Road, Hangzhou Zhejiang, 310003 People’s Republic of China; 2 Department of Radiology, the First Affiliated Hospital, College of Medicine, Zhejiang University, Zhejiang, Hangzhou 310003 China; 3 Department of Oncology, the First Affiliated Hospital, College of Medicine, Zhejiang University, 79 Qingchun Road, Zhejiang, Hangzhou 310003 China; 4 Department of Radiation Oncology, Henan Province People’s Hospital, Zhengzhou, Henan 450000 China

**Keywords:** Apparent diffusion coefficient value, Nasopharyngeal carcinoma, Diffusion-weighted magnetic resonance imaging

## Abstract

**Background:**

Diffusion-weighted MR imaging (DWI) has increasingly contributed to the management of nasopharyngeal carcinoma (NPC) patients. The objective of this paper was to explore the prognostic significance of apparent diffusion coefficient (ADC) values in 93 NPC patients.

**Methods:**

This retrospective study included 93 newly diagnosed NPC patients. Pretreatment ADC values were determined and compared with patients’ age, gender, alcohol intake, smoking, tumor volume, pathological type, tumor stage, and nodal stage. Using the Kaplan-Meier method, overall survival (OS), local relapse-free survival (LRFS), and distant metastasis-free survival (DMFS) were calculated and the values compared between the low and high ADC groups. Multivariate analysis of ADC values and other 9 clinical parameters was performed using a Cox proportional hazards model to test the independent significance for OS, LRFS and DMFS.

**Results:**

The mean ADC value for the initial nasopharyngeal tumors was 0.72 × 10^−3^ mm^2^/s (range: 0.48–0.97 × 10^−3^ mm^2^/s). There was no significant difference between pretreatment ADCs and patient’ gender, age, smoking, alcohol intake, or tumor stage. A significant difference in the ADCs for different N stages (*P* = 0.022) and correlation with initial tumor volume (*r* = −0.26, *P* = 0.012) were observed. In comparison, the ADC value for undifferentiated carcinoma was lower than that for other 3 pathological types. With a median follow-up period of 50 months, the 3-year and 5-year OS rates were 88.2% and 83.3%, respectively, 3-year and 5-year LRFS rates were 93.5% and 93.3%, respectively, and 3-year and 5-year DMFS rates were 83.9% and 83.3%, respectively. Patients with tumor ADC values ≥0.72 × 10^−3^ mm^2^/s exhibited longer OS and LRFS periods compared with tumor ADC values <0.72 × 10^−3^ mm^2^/s, with *P* values 0.036 and 0.018, respectively. In addition, patients with deaths or recurrences or distant metastasis had significant lower ADC values than those without disease failures. According to a multivariate analysis using the Cox proportional hazard test, ADC values showed a significant correlation with OS (*P* = 0.0004), LRFS (*P* = 0.0009), and DMFS (*P* < 0.0001), respectively.

**Conclusions:**

Pretreatment tumor ADC values supposed to be a noninvasive important prognostic parameter for NPC.

## Background

Nasopharyngeal carcinoma (NPC) is a head and neck malignancy commonly diagnosed in southern China and southeast Asia [1]. Moreover, the World Health Organization (WHO) estimates that over 80,000 new cases of NPC are diagnosed worldwide [1]. It is important to identify factors that are useful for predicting prognosis and helping personalize therapies. Established prognostic factors include histopathological type, tumor stage, and nodal stage. Furthermore, these factors have been shown to correlate significantly with the overall survival (OS) and progress-free survival (PFS) in NPC patients [2–5].

Magnetic resonance imaging (MRI) plays an important role in managing patients with NPC. For example, it is used for tumor staging, for delineating target volumes, and for detecting recurrence [6–8]. Another valuable imaging technique is diffusion-weighted MR imaging (DWI), and for its sensitivity to the motion of water molecules, it reflects the viability and structure of tissues on a cellular level [9, 10]. DWI is increasingly applied in the head and neck patient; for example, to distinguish recurrence and post-irradiation change. Moreover, DWI can differentiate metastatic lymph nodes from benign lymphadenopathy or nodal lymphomas [11–13], and DWI can also detect nodal and distant metastases [14, 15]. Furthermore, DWI is useful for monitoring the treatment response following chemotherapy or radiation [16].

Apparent diffusion coefficient (ADC) values have recently been reported to correlate with several prognostic parameters for varied tumors [17–19], such as retinoblastoma, lung cancer, breast cancer, and head and neck cancers [20, 21]. In this study, to explore whether similar results are obtained, we correlate tumoral ADC with treatment outcomes in a homogeneous group of NPC patients who exhibit a different pathogenesis, biological behavior, and natural course from other head and neck cancer patients.

## Methods

### Study patients

We retrospectively analyzed pretreatment MR-images and other clinical information from 93 consecutive newly diagnosed NPC patients. Endoscopic examinations to detect a clinically suspected lesion in the nasopharynx were performed on all patients, and pathology was obtained at first diagnosis. Distant metastases were ruled out during staging workup using chest computed tomography (CT), abdominal ultrasound, and bone scintigraphy. The ethical committee of Zhejiang University approved this analysis. Patient consents were obtained from all of the studied patients.

### MRI and DWI techniques

MRI was performed using a Philips 3.0 T Intera Master (Philips, Amsterdam, The Netherlands) with a standard head coil, two-channel dedicated surface neck coil, and spine coil. The transverse sequences consisted of 44 slices (5 mm each) and a 0.5 mm intersection gap. DWI was performed using a multiple section spin-echo single-shot echoplanar sequence in the transverse plane. A single-shot echoplanar sequence was also carried out before the injection of contrast agent gadolinium DTPA (Gd-DTPA), and this consisted of a 96 × 96 matrix, a TR/TE = 2947.1 ms/43.3 ms, b-values of 0 and 1500 s/mm^2^, a field-of-view (FOV) of 260 × 260 mm^2^, and a NSA of 6. To obtain the best image quality, an integrated phase correction was applied during DWI.

### Acquisition of ADC values

DWI data was analyzed by an experienced radiologist blinded to this study. A workstation (Agfa-Gevaert, Mortsel, Belgium) was used to identify a region of interest (ROI) for each definitive solid lesion, while avoiding necrotic or cystic components that were observed to be ≤10 mm^2^ with DWI. Subsequently, ADC values of ROIs were acquired from ADC maps directly, reconstructed using b values of 1500 and 0 s/mm^2^. ROIs were collected on 2 to 3 slices for every lesion to quantitate the primary tumor’ ADC and the tumor’s final ADC value was defined as an average value for these ROIs.

### Patient treatment

All 93 patients received radical intensity modulated radiation therapy (IMRT). Dose of 6540–7412 cGy/30–34F was delivered to each planned gross tumor volume (PGTV), while 5264 cGy/28F to 6016 cGy/32F was given to each planning target volume (PTV). A total of 88 patients received concurrent chemotherapy with platinum-based drugs (80 mg/m^2^) intravenously every 3 weeks for 3 courses during IMRT, while the other 5 patients received IMRT alone (either because of early tumor stage or they refused to receive chemotherapy).

### Clinical endpoint

Patient follow-ups were scheduled every 1 or 2 months within the first half year of a diagnosis, then every 3 months for the next 6 months, and once every 6 months thereafter. MRI with contrast enhancement and DWI were performed to evaluate locoregional recurrence. Chest CT, abdominal ultrasound, and bone scintigraphy, and less frequently positron emission tomography (PET)/CT, were also conducted to detect distant metastasis. Local relapse was established based on histologic confirmation (biopsy or surgical resection), detection of a new mass, or a serial increase in size of a residual mass. In addition, distant failure was determined with detection of any new masses in the liver, lung, bone, or brain during routine evaluations conducted during a follow-up period of at least 1 year. Overall survival (OS) was calculated from the completion of IMRT until death. Local relapse was defined based on primary tumor or regional lymph node recurrence, while distant failure was defined as distant metastasis.

### Statistical analysis

SAS v9.0 statistical software package was used for statistical analysis. In addition, mean ± standard deviation (SD) ADC values for each prognostic parameters were measured. Student’s t-test was applied to independent samples to identify differences in ADC values between two groups, while one-way analysis of variance (ANOVA) was used to evaluate differences between more than two groups. Pearson correlation was performed to correlate NPC ADC values with tumor volume and to correlate primary ADC values with ADC values for the cervical lymph nodes. *P*-values and *r* values were also calculated. Using the Kaplan-Meier method, patient survival (including OS, LRFS, and DMFS) were calculated and the values compared between the low and high ADC groups; differences were compared using the log-rank test. Independent significance of different factors was tested using multivariate analysis in a Cox proportional hazards model. When testing the association with survival (including OS, LRFS, DMFS), patient age, gender, smoking, alcohol intake, tumor volume, pathological type, tumor stage, nodal stage, and pretreatment ADCs were included in multivariate analyses. A *P* value of less than 0.05 was considered significant.

## Results

### Patient characteristics

The present cohort included 69 males and 24 females with a median age of 52 years (range: 22–82 years). According to the 7th edition of the American Joint Committee on Cancer (AJCC) manual, 3 patients had stage I disease, 19 patients had stage II, 55 were stage III, and 16 were stage IV (comprising 12 with IVa disease and 4 with IVb disease). Histologically, 30 lesions were identified as well-differentiated non-keratinizing carcinomas, 39 as poorly differentiated non-keratinizing, 8 as keratinizing squamous cell, and 16 as undifferentiated carcinomas (Table 1).

**Table 1:** Mean, minimum, and maximum ADC values for the NPC cases analyzed according to various clinical characteristics

Factors	N (Total = 93)	ADC values			*P*-value
		Minimum	Maximum	Mean ± SD	
Age					0.83
< 50 y	37	0.52	0.92	0.73 ± 0.11	
≥ 50 y	56	0.48	0.97	0.72 ± 0.10	
Gender					0.28
Male	69	0*.*48	0.97	0.72 ± 0.10	
Female	24	0.57	0.92	0.74 ± 0.10	
Smoking status					0.30
Yes	46	0.57	0.92	0.73 ± 0.09	
No	47	0.48	0.97	0.71 ± 0.11	
Alcohol intake					0.75
Yes	58	0.48	0.92	0.73 ± 0.09	
No	35	0.58	0.97	0.72 ± 0.11	
Pathological type*					0.51
1	8	0.61	0.97	0.79 ± 0.13	
2	39	0.57	0.96	0.72 ± 0.10	
3	30	0.48	0.97	0.72 ± 0.10	
4	16	0.59	0.83	0.70 ± 0.06	
Tumor stage					0.53
T1	15	0.48	0.92	0.71 ± 0.14	
T2	48	0.57	0.97	0.73 ± 0.09	
T3	17	0.59	0.95	0.74 ± 0.10	
T4	13	0.59	0.85	0.69 ± 0.07	
Nodal stage					0.022
N0	10	0.52	0.84	0.66 ± 0.08	
N1	16	0.47	0.97	0.72 ± 0.12	
N2	63	0.57	0.95	0.73 ± 0.09	
N3	4	0.64	0.97	0.81 ± 0.13	

*ADC*: apparent diffusion coefficient; *NPC*: nasopharyngeal carcinoma; *SD*: standard deviation

*Type1: keratinizing squamous cell carcinoma; Type 2: poorly differentiated non-keratinizing carcinoma; Type 3: well differentiated non-keratinizing carcinoma; Type 4: undifferentiated carcinoma

### Tumor ADC values and prognostic parameters

The mean ADC value for the primary tumors analyzed was 0.72 × 10^−3^ mm^2^/s, range: 0.48–0.97 × 10^−3^ mm^2^/s. In addition, Table 1 lists the minimum, maximum, and mean ADC values in correlation to patient age, gender, smoking and drinking status, tumor pathological type, tumor grade, and metastatic cervical lymph nodes. The most common histopathological type of NPC for this cohort was poorly differentiated non-keratinizing carcinoma (type 2, *n* = 39), followed by well differentiated non-keratinizing carcinoma (type 3, *n* = 30). The ADC values for keratinizing squamous cell carcinoma (type 1), poorly differentiated non-keratinizing carcinoma(type 2), and well differentiated non-keratinizing carcinoma (type 3) NPC were 0.79 ± 0.13 × 10^−3^ mm^2^/s, 0.72 ± 0.10 × 10^−3^ mm^2^/s, and 0.72 ± 0.10 × 10^−3^ mm^2^/s, respectively. In comparison, the ADC value for type 4 (undifferentiated carcinoma) was 0.70 ± 0.06 × 10^−3^ mm^2^/s, which was lower than that for types 1–3. However, between type 1 and type 4, there was a significant difference with a *P* value 0.024.

The most common tumor stage was T2 (*n* = 48), and the ADC values for T2 and T3 tumors (0.73 ± 0.09 × 10^−3^ mm^2^/s and 0.74 ± 0.10 × 10^−3^ mm^2^/s, respectively) were higher than those for T1 and T4 tumors (0.71 ± 0.14 × 10^−3^ mm^2^/s and 0.69 ± 0.07 × 10^−3^ mm^2^/s, respectively). However, no significant difference between the four groups were observed (*P* = 0.53).

According to metastatic cervical lymph node status, the mean ADC values for the primary tumors were 0.66 ± 0.08 × 10^−3^ mm^2^/s for patients with N0 (*n* = 10), 0.72 ± 0.12 × 10^−3^ mm^2^/s for N1 (*n* = 16), 0.73 ± 0.09 × 10^−3^ mm^2^/s for N2 (*n* = 63), and 0.81 ± 0.13 × 10^−3^ mm^2^/s for N3 (n = 4). Furthermore, the ADC values did significantly differ between these N staging groups (*P* = 0.022) (Table 1).

**Fig. 1:**
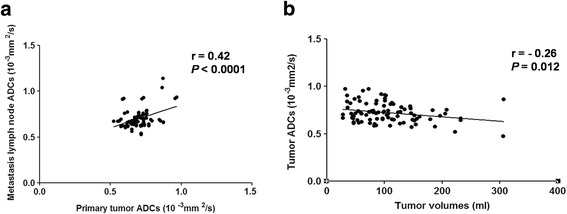
Pearson correlations between pretreatment tumor apparent diffusion coefficient (ADC) values and lymph node ADC values (**a**), and between tumor volume and pretreatment tumor ADC values (**b**). ADC: apparent diffusion coefficient

The mean ADC value for the metastatic cervical lymph nodes was 0.70 ± 0.095 × 10^−3^ mm^2^/s, and an obvious positive correlation was observed between the ADC values for primary tumors and the ADC values for metastatic cervical lymph nodes (*r* = 0.42, *P* < 0.001) (Fig. 1). The median tumor volume (including both primary nasopharyngeal tumors and metastatic cervical lymph nodes) was 85.5 ml (range, 21.8–306 ml), and the primary tumor ADC values were found to negatively correlate with tumor volume (*r* = −0.26, *P* = 0.012). Consequently, lower ADC values were found to represent larger tumor volumes (Fig. 1).

### Tumor ADC values and survival outcomes

The median duration of the follow-up period following the completion of radiotherapy was 50 months (range, 36–68 months). During this time, 20/93 patients died, 3 due to fatal nasopharyngeal bleeding (caused by tumor invasion) and the remaining 17 due to distant tumor failure. The 3- and 5-year OS rates were 88.2% (88/93) and 83.3% (25/30) and the median OS period was 46 months (range, 5–68 months). 9 patients experienced local relapse, which included 5 with nasopharyngeal primary tumor relapse, 2 with retropharyngeal lymph node (RLN) recurrence, and 2 with cervical regional lymph node relapse.

The 3- and 5-year LRFS rates were 93.5% (87/93) and 93.3% (28/30), and the median recurrence time was 44 months. Distant metastasis developed in 23/93 patients, including 8 cases with hepatic metastasis, 3 with pulmonary metastasis, 5 with bone metastasis, 1 with retroperitoneal metastasis, and 6 with poly-organ metastasis. Moreover, the 3- and 5-year DMFS rates in the present study were 83.9% (78/93) and 83.3% (25/30), respectively; the median distant failure time was 8 months. For patients with tumor ADC values <0.72 × 10^−3^ mm^2^/s (e.g., the low ADC group, lower than mean tumor ADC values): the 3-year OS rate was 84% (42/50), the median OS period was 45 months, the 3-year LRFS rate was 88% (44/50), and the 3-year DMFS rate was 82% (41/50). For patients with tumor ADC values ≥0.72 × 10^−3^ mm^2^/s (e.g., the high ADC group): the 3-year OS rate was 93% (40/43), the median OS period was 60 months, the 3-year LRFS rate was 97.7% (42/43), and the 3-year DMFS rate was 86% (37/43).

**Fig. 2:**
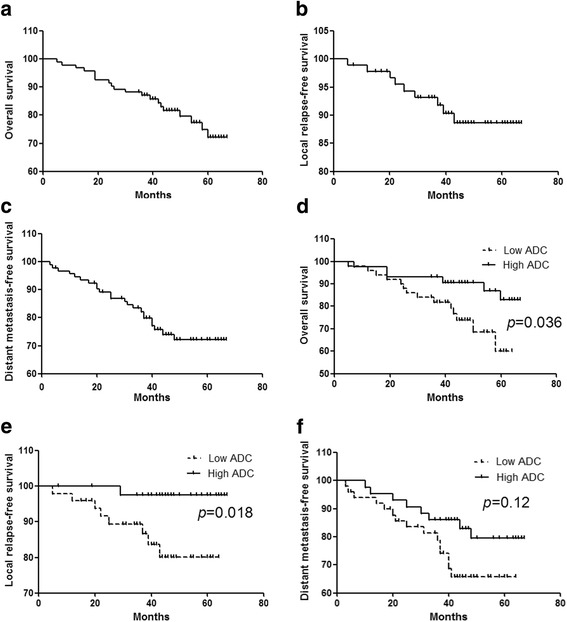
Kaplan-Meier (**a**) OS curves, (**b**) LRFS curves, and (**c**) DMFS curves. In addition, (**d**) OS, (**e**) LRFS, and (**f**) DMFS curves were compared for the low ADC group (*dashed line*) and the high ADC group (*solid line*). OS: overall survival; LRFS: local relapse-free survival; DMFS: distant metastasis-free survival; ADC: apparent diffusion coefficient

Most deaths (14/20) and recurrences (9/10), as well as most of the distant metastasis events (15/23), occurred in the low ADC group. Kaplan-Meier survival data are presented in Fig. 2. Patients in the low ADC group exhibited a significant difference in OS and LRFS compared with the high ADC group (*P* = 0.036, *P* = 0.018). Moreover, while DMFS periods for the high ADC group appeared to be longer than those for the low ADC group, but the difference was not indicated significant (*P* = 0.12) (Fig. 2).

ADC showed a significant correlation with OS (*P* = 0.0004), LRFS (*P* = 0.0009), DMFS (*P* < 0.0001), respectively, according to the multivariate analysis using the Cox proportional hazard test (Table 2). Results demonstrated that pretreatment ADC was an independent prognostic parameter for survival. In addition, clinical stage and N stage were independent prognostic parameters for OS (*P* = 0.0066 and 0.0203, respectively), and DMFS (*P* = 0.006 and 0.0337, respectively) (Table 2).

**Table 2:** Multivariate analyses of prognostic factors in the 93 NPC patients

	OS		LRFS		DMFS	
	*P* value	95% CI	*P* value	95% CI	*P* value	95% CI
ADC	*0.0004*	0.001–0.13	*0.0009*	0–0.095	*0.0001*	0–0.023
Sex	0.17	0.34–1.21	0.45	0.38–1.54	0.20	0.35–1.25
Age	0.72	0.97–1.02	0.85	0.97–1.02	0.13	0.96–1.005
Clinical stage	*0.006*	1.25–3.95	0.19	0.81–3.02	*0.006*	1.26–4.01
T stage	0.076	0.49–1.04	0.61	0.59–1.36	0.11	0.93–1.93
N stage	*0.02*	0.35–0.92	0.11	0.36–1.10	*0.034*	0.36–0.96
Pathological type	0.61	0.73–1.20	0.30	0.62–1.16	0.36	0.67–1.16
Tumor volume	0.69	0.996–1.007	0.38	0.99–1.01	0.15	0.99–1.01
Smoking	0.60	0.46–1.56	0.95	0.50–2.10	0.80	0.50–1.69
Drinking	0.57	0.66–2.13	0.74	0.44–1.78	0.10	0.31–1.11

*ADC*: apparent diffusion coefficient; *CI*: confident index; *NPC*: nasopharyngeal carcinoma; *OS*: overall survival; *LRFS*: local relapse-free survival; *DMFS*: distant metastasis-free survival; *p*<0.05 as statistically significant

## Discussion

In this study, patient characteristics (such as age, gender, smoking, and drinking) showed no relationship with ADC values. But the results were similar to those of previous reports in which ADC values were found to correlate with different histologic types of carcinomas [22]. Moreover, Razek et al. reported a significant association between ADC values and the degree of tumor differentiation for retinoblastomas [19]. NPC includes nonkeratinizing carcinomas (both differentiated and undifferentiated), keratinizing squamous cell carcinoma (SCC), and basaloid SCC. Furthermore, the most common histologic type of NPC is nonkeratinizing carcinomas, consist of 75–99%. The characteristic of this type tumor is comprised tableted of concentrated carcinoma cells separated by an infiltrating of plasma cells and lymphocytes [23].

In this research, the highest mean ADC value was associated with type 1 NPC (e.g., keratinizing squamous cell carcinoma). Conversely, the lowest ADC value was associated with type 4 NPC (e.g., undifferentiated carcinomas). However, while there was a significant difference between these two types (*P* = 0.024), no significant difference was found among the other two histopathological types. Driessen et al., in a prospective study of 17 head and neck SCC [20], reported similar results-they found no obvious correlation between tumor ADC and tumor histologic grade, however a trend was found that poorly differentiated tumors had lower ADC values in comparison with moderately or well differentiated tumors.

Insufficient sample size may have limited our ability to obtain more significant results, or this result may suggest that ADC values partly reflect the differentiation of NPCs. However, a significant correlation with the histological type and mean ADC value was found among 27 cases of breast cancer in a study by Yoshikawa et al. [24]. Undifferentiated carcinomas reportedly predict the worst prognosis [3]. Results in the present study suggest that low pretreatment tumor ADC was a poor prognostic factor. In addition, necrosis should be considered a critical parameter. Even when we delineated ROI in this study as little as possible to avoid containing obvious necrosis, micronecrosis would still exist in minor ROI. Furthermore, it is known that necrosis leads to high ADC values [25]. Squamous cell carcinoma or well differentiated tumors may contain much more necrosis than undifferentiated carcinomas and thus favor high ADC values.

In the present study, we hypothesized a negative relevance between ADC values and prognostic factors reflecting mitosis (such as tumor stage, lymph node stage, and tumor volume). Interestingly, for both tumor and regional lymph node grading, only the latter was found to significantly correlate with the mean ADC values obtained. This may be due to tumor staging in relation to the patterns of spread for NPC. For example, in some patients, the tumor may invade bony structures or intracranial tissues and/or cranial nerves by superior spread, even though the tumor volume may be small and a low lymph node staging is obtained. Furthermore, these patients are diagnosed high T stage.

When the relationship between different N stage was investigated, similar results to those reported by Razek et al. were obtained, with the ADC values being significantly lower for positive metastatic cervical lymph nodes compared with negative metastatic cervical lymph nodes [26]. In another study by Razek et al. [18], significant differences were observed in the ADC values for lung cancer cases involving N0 and N3 lymph nodes (*P* = 0.043). Similarly, a positive relation was observed between primary tumor ADC values and ADC values for metastatic cervical lymph nodes (*r* = 0.17, *P* < 0.001).

Taken together, these results suggest that primary tumors and metastatic cervical lymph nodes are homogeneous and may exhibit similar biological behaviors. Furthermore, this study suggests that ADC can reflect N stage more sensitive than T stage. Recent studies increasingly have raised proposals for revisions in the following edition of TNM staging system in NPC [27–30]. Some even suggested take new biomarkers such as epstein-barr virus (EBV) DNA or miRNA into account in the staging system since these biomarkers reportedly have prognostic value as well [29, 30]. Thus, as another prognostic value, ADC value should be taken into account in the new TNM staging system.

Primary NPC tumor volume reportedly is an important independent prognostic factor in NPC patients [31, 32]. For instance, in the 2011 study by Chen et al. [32], patient had a poor 5-year OS in the group with tumor volume > 50 ml, indicated that large tumor volume is almost equivalent to the T4 stage. A large tumor volume usually exhibits a greater metastatic potentiality, and therefore, is correlated with a poorer prognosis. Previously, a negative correlation between ADC values according to tumor size was identified for breast cancers (*r* = −0.504, *P* = 0.001), retinoblastomas (*r* = −0.680, *P* = 0.015), and NPCs (*r* = −0.799, *P* = 0.03) [17, 19, 26]. Similarly, a reversed correlation was observed between tumor volumes and ADC values in the present study (*r* = −0.26, *P* = 0.012). This may be explained by the observation that larger tumors are generally more restricted in their diffusion, are usually poorly differentiated, or represent an undifferentiated malignancy.

Performance status associated with local control, disease-free, and overall survival was reported in head and neck SCC and NPC in studies performed in Japan and China [33, 34]. In the present study, we defined the mean ADC value (0.72 × 10^−3^ mm^2^/s) as the threshold level. Hence the high ADC group was higher than or equal to the mean level and the low ADC group was lower than the mean level. Results demonstrated the high ADC group was correlated with a longer OS period and LRFS period in NPC, and with a significant difference (*P* = 0.036 and 0.018, respectively). Different threshold options would likely give different results. Furthermore, a significant correlation between ADC with long-term outcomes was also observed, with the *P* values for OS, LRFS, and DMFS being 0.0004, 0.0009, and <0.0001, respectively. Thus, the pretreatment ADC value should be take into a consideration of a prognostic factor in NPC.

The present study has some limitations. First, due to the most patients are locoregionally advanced cases, patient selection bias may exist. Second, only two b values were used in this study for ADC measurement, so the ADC measurement may be insufficiently reliable. Third, this study was only performed at one center and was comparatively homogeneous, further multicenter and large-scale studies are required to strengthen the findings.

## Conclusions

This study revealed that ADC values correlated with prognostic parameters of NPC. Specifically, a low ADC value was demonstrated to have correlation with undifferentiated tumors, a larger tumor volume, and metastatic lymph node stage. Incorporating the pretreatment ADC value in the future clinical staging system is challenging. Moreover, further studies, especially multicenter and prospective studies, are required to confirm the observation of the present study that low pretreatment tumor ADC values predict a poor prognosis for NPC patients.

## Abbreviations

ADC: Apparent diffusion coefficient
AJCC: American Joint Committee on Cancer
ANOVA: One-way analysis of variance
CT: Computed tomography
DMFS: Distant metastasis-free survival
DWI: Diffusion-weighted MR imaging
EBV: Epstein-barr virus
LRFS: Local relapse-free survival
MRI: Magnetic resonance imaging
NPC: Nasopharyngeal carcinoma
OS: Overall survival
PET: Positron emission tomography
PFS: Progress-free survival
PGTV: Planned gross tumor volume
PTV: Planning target volume
RLN: Retropharyngeal lymph node
ROI: Region of interest
SCC: Squamous cell carcinoma
SIB-IMRT: Simultaneous integrated boost intensity modulated radiation therapy
WHO: World Health Organization
